# β Integrins Mediate FAK Y397 Autophosphorylation of Resistance Arteries during Eutrophic Inward Remodeling in Hypertension

**DOI:** 10.1159/000365479

**Published:** 2014-10-09

**Authors:** Egidius H.J Heerkens, Lisa Quinn, Sarah B Withers, Anthony M Heagerty

**Affiliations:** Institute of Cardiovascular Sciences, University of Manchester, Manchester, UK

**Keywords:** Integrins, Resistance arteries, Eutrophic remodeling, Hypertension

## Abstract

Human essential hypertension is characterized by eutrophic inward remodeling of the resistance arteries with little evidence of hypertrophy. Upregulation of αVβ3 integrin is crucial during this process. In order to investigate the role of focal adhesion kinase (FAK) activation in this process, the level of FAK Y397 autophosphorylation was studied in small blood vessels from young TGR(mRen2)27 animals as blood pressure rose and eutrophic inward remodeling took place. Between weeks 4 and 5, this process was completed and accompanied by a significant increase in FAK phosphorylation compared with normotensive control animals. Phosphorylated (p)FAK Y397 was coimmunoprecipitated with both β1- and β3-integrin-specific antibodies. In contrast, only a fraction (<10-fold) was coprecipitated with the β3 integrin subunit in control vessels. Inhibition of eutrophic remodeling by cRGDfV treatment of TGR(mRen2)27 rats resulted in the development of smooth-muscle-cell hypertrophy and a significant further enhancement of FAK Y397 phosphorylation, but this time with exclusive coassociation of pFAK Y397 with integrin β1. We established that phosphorylation of FAK Y397 with association with β1 and β3 integrins occurs with pressure-induced eutrophic remodeling. Inhibiting this process leads to an adaptive hypertrophic vascular response induced by a distinct β1-mediated FAK phosphorylation pattern.

## Introduction

The resistance arteries (<300 μm in internal diameter) are essential for mediating the autoregulation of flow and the stabilization of capillary pressure. An important component of this protective mechanism, called the myogenic response, allows these vessels to change diameter in response to alterations in intraluminar pressure. Besides resulting in enhanced myogenicity of the resistance arteries [1], sustained high blood pressure is associated with changes in cardiovascular structure. Resistance vessels develop a narrowed lumen and a thickened vascular wall, but without an apparent increase in cross-sectional area. This process, known as inward eutrophic remodeling, occurs as a response to prolonged (myogenic) constriction in order to protect the downstream vasculature, and it is thought to be an energetically favored mechanism to preserve a reduced lumen diameter for long periods. Such a structural adaptation involves a migratory process, whereby vascular smooth-muscle cells (VSMCs) reposition in the vascular wall [2], and it can be observed in most forms of hypertension including onset and milder hypertensive states. When hypertrophy is observed, the rise in blood pressure is often fulminant and severe. Autoregulatory mechanisms are then inadequate to normalize wall stress. Recently, it has been reported that the development of small-artery hypertrophy is an adverse prognostic sign [3].

The integrin/extracellular-matrix (ECM) axis transfers tensile forces exerted by blood pressure across the cell membrane. Integrins are cell surface ECM receptors that can heterodimerize in a noncovalent fashion to form 24 different αβ receptors which recognize a wide variety of cellular and ECM components present in the arterial extracellular space including collagen, fibronectin and laminin [4]. In resistance arteries, specific integrin subtypes are initially utilized for the mechanotransduction of pressure [5], while others mediate the migration of VSMCs towards a narrowed lumen [6,7]. Engagement of integrins upon migration activates intracellular signaling complexes found at focal adhesion (FA) sites [8]. To date, there has been no conclusive evidence that FA sites, which contain active tyrosine kinases (e.g. focal adhesion kinase, FAK) and other cytoskeletal associated phosphoproteins observed in migrating cells in vitro [9], exist in vivo in arterial VSMCs. However, it is clear that the migratory process of arterial VSMCs in vivo is more subtle and is limited to the elongation of tapered cells and an increase in cell overlap [2] compared with the more pronounced movement of cells in vitro. Eutrophic remodeling is a relatively rapid process that is followed by the fixation of cells within the vascular ECM scaffold and it requires active transglutaminases [10]. Recently, we described a key role for αVβ3 integrin in eutrophic remodeling and other studies have described its function in myogenic constriction [6].

There is increasing evidence that FAK is an important mediator for cell attachment and mechanosensing [11]. The Src family kinases are also regarded as key components of mechanotransduction to special domains via the cytoskeleton when force is applied to fibronectin-binding integrins such as αVβ3 [12]. Moreover, FAK/Src association with specific integrin subunits seems to regulate particular cellular functions: for example, α5β1 appears to regulate the L-type calcium current, which mediates myogenic constriction via Src and FAK signaling [13], whereas the association of FAK/Src with β3 integrin subunits seems to be important for cell migration [14]. In addition, evidence is accumulating for the initiation of FAK phosphorylation in the growth response of arteries exposed to elevated intraluminal pressure [15].

We wished to test the hypothesis that FAK/Src signaling is crucial in mechanotransduction and the eutrophic inward remodeling of resistance arteries as a response to elevated pressure.

This study examined the activation levels of FAK/Src in arteries exposed to elevated pressure in vitro and ex vivo, and we report differential FAK Y397 phosphorylation levels present at the integrin β subunits of resistance arteries from hypertensive TGR(mRen2)27 rats as pressure rises and eutrophic inward remodeling occurs. Ex vivo pressurization of arterial segments which exhibit myogenic tone confirmed that FAK Y397 phosphorylation is increased as a response to elevated pressure.

## Methods

### Animals

The TGR(mRen2)27 rat carries a DBA/2J Ren2 transgene and develops hypertension with vascular structural changes from eutrophic inward remodeling [6,16]. These animals, together with Sprague-Dawley (SD) normotensive control animals, were studied by immunohistochemistry and Western blot at 4 and 5 weeks of age. Ex vivo studies were performed on arteries harvested from SD rats at 5 weeks of age. On the day of study, rats were sacrificed by stunning and cervical dislocation. All animal procedures were performed in accordance with the UK Home Office Regulated Procedures on Living Animals (Scientific Procedures) Act 1986.

### Blood Pressure Measurement

Blood pressure measurements of TGR(mRen2)27 and SD control animals were performed using tail-cuff plethysmography under light fluothane anesthesia.

### Vessel Morphology and Integrin Antagonism

We performed studies on the small arteries harvested from animals sacrificed at 5 weeks of age, because hypertension-induced eutrophic remodeling takes place from the age of 4 weeks and is completed by week 5, as described previously [6]. Segments of 2nd-order arteries (approx. 200 µm) were isolated from the proximal region of the mesenteric bed, mounted on a wire myograph and held in physiological salt solution (PSS; 119 mM NaCl, 4.7 mM KCl, 25 mM NaHCO_3_, 1.17 mM KH_2_PO_4_, 1.17 mM MgSO_4_, 0.026 mM EDTA, 1.6 mM CaCl_2_ and 5.5 mM glucose) at 37°C and gassed with 5% CO_2_ and 95% O_2_ to maintain a pH of 7.4. Subsequent morphological parameters of live vessels such as the media-to-lumen ratio, growth and remodeling indices were then measured and calculated, as previously described [17].

In order to study the effects of integrin αV antagonism, TGR(mRen2)27 rats were injected intraperitoneally at the age of 4 weeks with cRGDfV or cRADfV peptide (10 mg/kg; Calbiochem) twice daily for 5 days before sacrifice [6]. These act as an αV integrin-specific inhibitory peptide, thereby inhibiting integrin functions or inactive control peptide, respectively [18,19,20]. Arteries were then harvested for morphological measurements using wire myography.

### Phosphorylated FAK Y397 Western Blot Analysis

Western blot analysis was performed according to the methodology described by Laemmli [21]. Vessels were dissected and protein extracted on ice in radioimmunoprecipitation assay buffer containing phosphatase and protease inhibitors to prevent changes in the phosphorylation status of the proteins under investigation. Anti-phosphorylated (p)FAK Y397 antibody (1:1,000, 0.5 μg/ml; AB4803, Abcam, Nottingham, UK), with a total (pan)-FAK antibody as a control for loading (1:1,000, 1.0 µg/ml; AB1311, Abcam), was used to detect expression in 25 µg of total protein. pFAK Y397 expression levels were corrected for the amount of total (pan)-FAK. Densitometric analysis was performed on a BioRad-GS690 scanner.

### Integrin αV Immunoprecipitation

An amount of 5 μg of each antibody was used to precipitate integrin subunits from 250 μg of total resistance artery radioimmunoprecipitation assay extract. The antibodies used for immunoprecipitation were: β1 (AB1952), β3 (AB1932) and α5 (aba5b) integrin antibodies (Chemi-Con, Moses Lake, Wash., USA). Agarose IgG/protein A (Sigma-Aldrich, UK) was used to bind and identify coprecipitated complexes. Subsequent Western blot analysis was performed using FAK Y397 (1:1,000) antibody and Src Y418 (1:500, 0.5 μg/ml; Abcam). Protein A-HRP was used for chemiluminescent detection of coprecipitated proteins [22].

### Ex vivo Pressure Arteriography

Arterial segments from the proximal region of the cremasteric artery from SD animals were dissected and mounted between two glass microcannulae in physiological salt solution in a pressure myograph (Living Systems Instrumentation, Burlington, Vt., USA). Experiments were carried out at 32°C. PP2 and PP3, i.e. a potent inhibitor of c-Src and an inactive analog (negative control) of PP2, respectively [23,24], were used at 1 μM (Calbiochem, Cambridge, UK). Only arteries that exhibited pressure-mediated constriction were included in the study. After pressurization at either 60 or 120 mm Hg for 1 h, arteries were quickly fixed in ice-cold 4% paraformaldehyde solution, left overnight, washed in 70% ethanol and prepared for histology/immunofluorescence. Immunohistochemical preparations of arteries ex vivo and in vitro were the same.

### Immunofluorescence Localization

Vessels for immunofluorescence were dissected on ice and snap-fixed in ice-cold 4% paraformaldehyde. Paraffin-embedded arteries were sliced at 4 μm, dewaxed and rehydrated. The pFAK Y397 integrin (1:100; AB4803, Abcam) and a nonimmune control IgG antibody were used separately, followed by donkey anti-rabbit antibody with conjugated TRITC (1:200, 1 μg/ml; Jackson ImmunResearch Laboratories, West Grove, Pa., USA). Diamidino-2-phenylindole (DAPI, 1 pg/ml) (Jackson ImmunoResearch Laboratories) was used as a blue fluorescent nuclear stain. A Leica DM6000 epifluorescence microscope was used at ×1,000 magnification in conjunction with Leica FW4000 software for image capture and analysis. Superimposing fluorescence and phase-contrast microscopy images allowed the visualization of the VSMC membrane and the internal elastic lamina which indicates the smooth-muscle/endothelium boundary.

### Statistical Analysis

Statistical analysis was performed using MS Excel. Values are expressed as mean ± standard deviation. Differences between data were tested using the two-tailed unpaired homoscedastic Student t test. p < 0.05 was considered statistically significant.

## Results

### Blood Pressure

Between 4 and 5 weeks of age, mean systolic blood pressure in TGR(mRen2)27 rats was significantly increased from 121 ± 7.9 to 148 ± 3.0 mm Hg (n ≥ 6, p < 0.02). SD control rats showed no age-dependent increase in blood pressure which ranged from 80.9 ± 8.8 to 93.4 ± 8.1 mm Hg. Pressures in TGR(mRen2)27 rats were significantly higher than those in SD rats at both time points (p < 0.0001).

The administration of cRGDfV and cRADfV peptides had no effect on the blood pressure rise seen in TGR(mRen2)27 rats (data not shown).

### Vascular Morphology

The cross-sectional area of TGR(mRen2)27 rat arteries at 5 weeks was unchanged compared with at 4 weeks of age, with a remodeling index of 97% indicating the development of eutrophic inward remodeling due to the media-to-lumen ratio being significantly increased (p < 0.01). The structural parameters of SD rat arteries did not change over 7 days.

cRGDfV treatment reduced the remodeling index to 9% and there was evidence of hypertrophic growth, with a calculated growth index of 17%. Treatment with cRADfV had no effect on remodeling, as predicted (data not shown).

### Western Blot

**Fig. 1:**
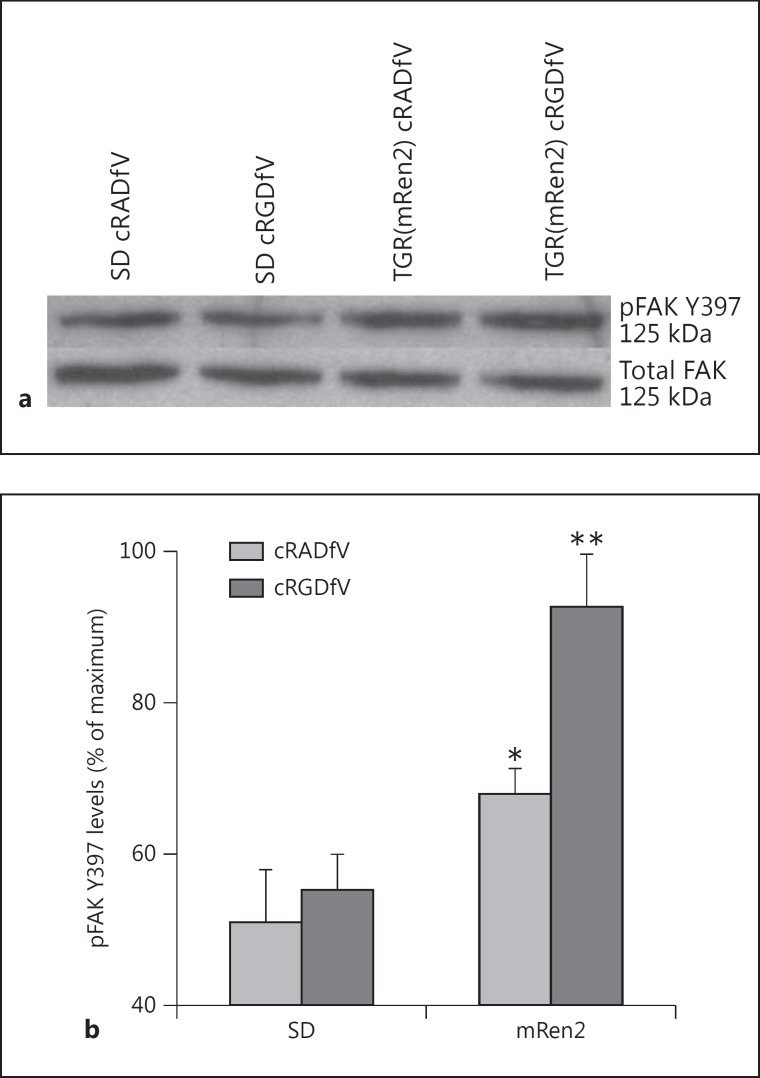
FAK Y397 phosphorylation in the resistance arteries of TGR(mRen2)27 rats treated with cRGDfV. **a** Representative Western blot of pFAK Y397 and total FAK (125 kDa) using 25 μg of protein from resistance artery extracts. **b** Levels of pFAK Y397 analyzed by densitometric scans and corrected for total FAK (n ≥ 4, * p < 0.05, ** p < 0.01).

Western blot analysis of pFAK Y397 showed significant rises in autophosphorylation levels in resistance arteries from TGR(mRen2)27 animals treated with cRADfV control peptide (approx. 1.4-fold, p < 0.05), compared with vessels from SD normotensive control animals (fig. 1a, b). Surprisingly, an increase in pFAK of approximately 2-fold was observed in TGR(mRen2)27 rat arteries treated with the active cRGDfV peptide (p < 0.01) compared with vessels from normotensive animals treated with the same peptide (n ≥ 4). Relative pFAK Y397 levels, established by densitometry, were corrected by comparison to total (pan)-FAK (fig. 1a, b).

### Immunofluorescence

**Fig. 2:**
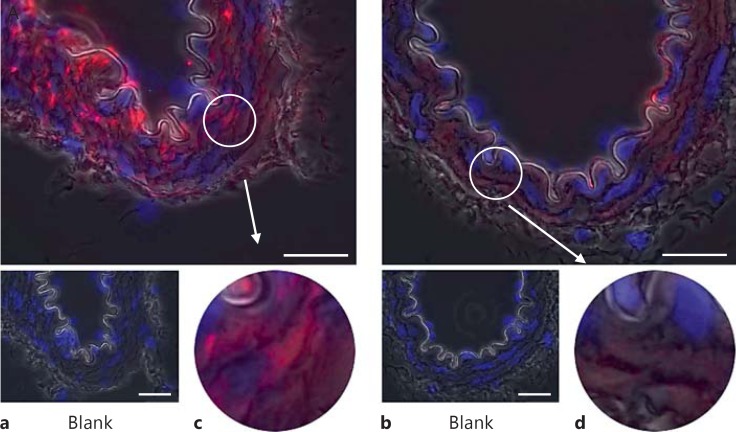
Basal levels of pFAK Y397 immunofluorescence in resistance mesenteric arteries. **a**, **c** TGR(mRen2)27 rat mesenteric artery and the prominent localization of pFAK Y397 to VSMCs. **b**, **d** pFAK Y397 localization in SD rat resistance arteries is loosely distributed throughout the cells in contrast with TGR(mRen2)27 rat arteries. ×1,000. Scale bar: 25 µm.

**Fig. 3:**
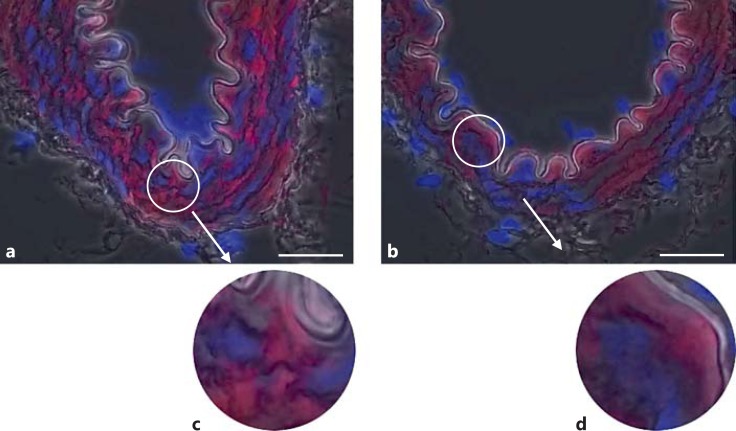
Total FAK (nonphosphorylated) immunofluorescence in resistance arteries. **a**, **c** The localization of total FAK throughout the VSMCs of TGR(mRen2)27 rat mesenteric artery. **b**, **d** Similarly, total FAK is present throughout the VSMCs of SD rat mesenteric arteries. ×1,000. Scale bar: 25 μm.

**Fig. 4:**
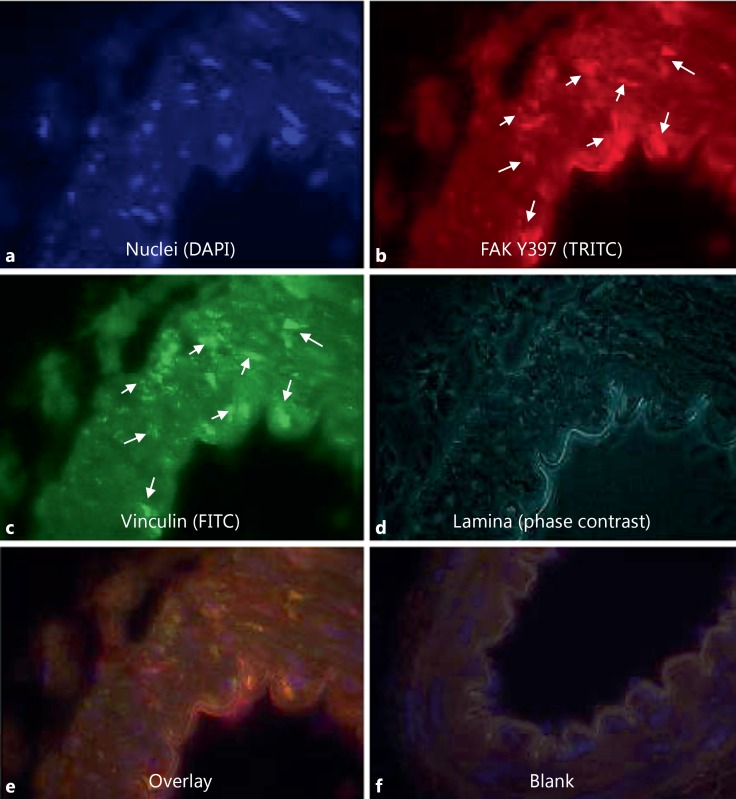
Localization of FAK Y397 at FA sites in SD rat resistance arteries. **a** DAPI staining for nuclei of within the resistance artery. FAK Y397 (**b**), vinculin staining (**c**) and phase-contrast images of the elastic lamina (**d**) demonstrate colocalization of FAK Y397 to FA sites within the medial layer of the artery (**e**). **f** A blank control.

**Fig. 5:**
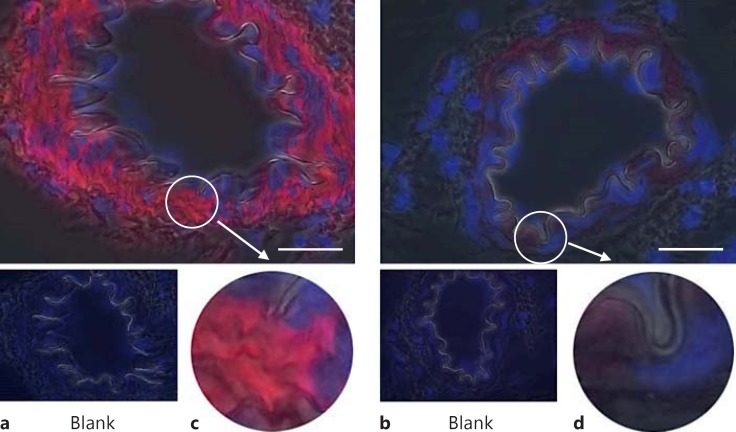
pFAK Y397 immunofluorescence of rat mesenteric arteries following α5 antagonism by cRGDfV treatment (10 mg/kg, intraperitoneal injection twice daily for 5 days). **a**, **c** pFAK Y397 staining is increased and present throughout the VSMC medial layer of TGR(mRen2)27 rat resistance arteries. **b**, **d** In contrast, cRGDfV treatment has no effect on pFAK Y397 levels in SD rat mesenteric arteries. ×1,000. Scale bar: 25 μm.

Phospho-FAK Y397 immunofluorescent staining of the arteries from normotensive SD animals was faint but present throughout the medial smooth-muscle cells (n ≥3; fig. 2b, d). The level of pFAK Y397 was observed to be increased in the VSMCs of TGR(mRen2)27 rat (n ≥ 3) resistance arteries (fig. 2a). Superimposing phase-contrast images onto fluorescent signals allowed the visualization of the VSMC medial layer, the internal elastic lamina and intense fluorescent pFAK Y397 regions of staining present in TGR(mRen2)27 rat VSMCs (fig. 2c). This is in contrast with fluorescence of total (nonphosphorylated) FAK (fig. 3a–d), which is expressed diffusely throughout the VSMCs of the medial layer (fig. 3c). FAK was localized to FA sites as identified by coimmunohistochemistry for FAK Y397 and vinculin, a membrane protein found associated with the FA sites of the elastic lamina (fig. 4). Control incubations with nonimmune IgG did not result in any detectable signal (fig. 2, 3, 4). The treatment of animals with the cRGDfV peptide (10 mg/kg) showed localization of pFAK within the medial layer of VSMCs and more intense staining in the treated TGR(mRen)27 rat arteries than in those from SD rats (n ≥ 3; fig. 5a) and more diffuse than in untreated arteries (fig. 2a), thereby supporting quantified data from our observations from Western blot analysis (fig. 1). Figure 5 a, c clearly shows intense Y397 staining is throughout the medial layer of arteries. In contrast, cRGDfV treatment of normotensive SD animals did not have any effect on pFAK Y397 levels (fig. 5b, d).

**Fig. 6:**
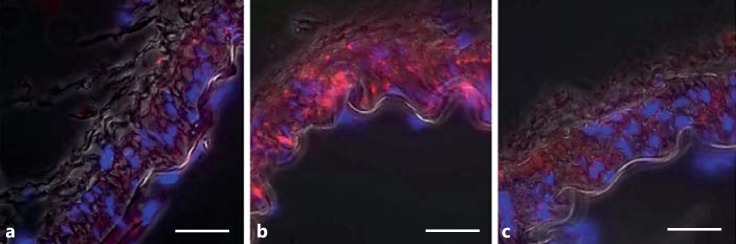
Immunofluorescence localization of phosphorylated FAK Y397 after ex vivo pressurization (60 and 120 mm Hg) of cremaster arteries. **a**, **b** PP3 coincubation has no effect on pressure-induced myogenic constriction or phosphorylation levels of FAK Y397. **a** Arteries at 60 mm Hg only present minimal phosphorylation of FAK Y397. **b** Arteries pressurized at 120 mm Hg exhibit prominent phosphorylation levels of FAK Y397. **c** Phosphorylation of FAK Y397 was abrogated when arteries were incubated with 1 µM PP2. Scale bar: 25 µm.

Inspection of arterial segments undergoing FAK Y397 phosphorylation staining, which were pressurized at 120 mm Hg for 1 h (and then developed myogenic-tone ex vivo) exhibited increases in FAK Y397 phosphorylation compared with vessels pressurized at 60 mm Hg (n ≥ 5). No passive morphological alterations of vessels to pressure were observed at this stage (data not shown). Phosphorylation of FAK Y397 at 120 mm Hg was abrogated when arteries were coincubated with 1 μM of PP2. In contrast, coincubation of PP3 did not have any effect (fig. 6).

### Integrin Coimmunoprecipitation

**Fig. 7:**
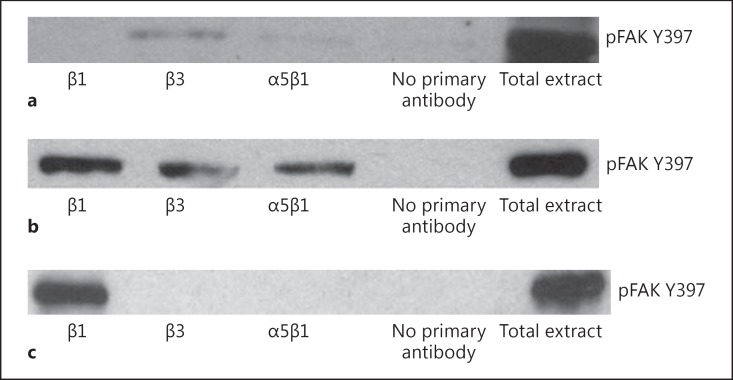
Integrin immunoprecipitation: coprecipitation of pFAK Y397. **a** pFAK Y397 is coprecipitated with β3 and α5β1 integrins in SD rat arteries. **b** In contrast, pFAK Y397 is coprecipitated with all the integrins tested in TGR(mRen2)27 rat arteries. **c** Inhibition of remodeling with cRGDfV peptides results in coprecipitation of pFAK Y397 only with β1 integrins. Immunoprecipitations shown are representative of at least 3 experiments.

pFAK Y397 was coprecipitated with integrin β3 and, to a lesser extent, with integrin α5β1 (fig. 7a) from the protein extracts of SD rat arteries. However, no FAK was coprecipitated with the general subpopulation of β1 integrins (fig. 7a).

In contrast to the protein extracts of normotensive SD resistance arteries, pFAK Y397 coprecipitation with all the integrins, i.e. β1, β3 and α5β1, was observed in the arteries of TGR(mRen2)27 animals (fig. 7b). Coimmunoprecipitation of the protein extracts from resistance arteries of 5-week-old cRGDfV-treated TGR(mRen2)27 animals (which inhibited eutrophic inward remodeling but showed hypertrophy) did not show pFAK cobinding with β3 or α5β1 integrins, but interacted solely with other subpopulations of β1 integrins (fig. 7c).

**Fig. 8:**
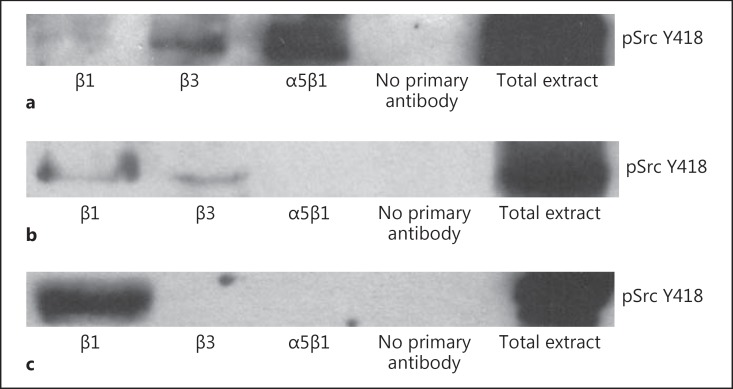
Integrin immunoprecipitation: coprecipitation of pSrc Y418. **a** pFAK pSrc Y418 is coprecipitated with β3 and α5β1 integrins in SD rat arteries. **b** In TGR(mRen2)27 rats, pSrc Y418 is coprecipitated with β1 and β3 integrins. **c** In contrast, cRGDfV treatment results in coprecipitation of pSrc Y418 with β1 integrins only. Immunoprecipitations shown are representative of at least 3 experiments.

In conjunction with FAK, c-Src was prominently coprecipitated with integrin α5β1 and, to a lesser extent, with integrin β3 (fig. 8a). In contrast, c-Src is not coprecipitated with the α5β1 integrins of resistance arteries of hypertensive TGR(mRen2)27 animals, but with integrins β1 and β3 (fig. 8b). c-Src, however is not recruited to the β3 cytoplasmic domain when αVβ3 binding to the ECM with the cRGDfV peptide is inhibited (fig. 8c).

## Discussion

Previously, we showed that the hypertension-mediated inward eutrophic remodeling of TGR(mRen2)27 rat resistance arteries commenced at 4 weeks of age and was complete at 5 weeks [6]. This process was dependent on the integrin αVβ3 (the only VSMC β3 integrin) because administration of cRGDfV peptide, which preferentially interferes with the binding of this integrin-heterodimer to the ECM [18,19,20,25], prevented the remodeling process but enhanced hypertrophy. We also investigated the localization of FAK and its activation status in the smooth-muscle cells of TGR(mRen2)27 rat resistance arteries and determined its association with β1 or β3 integrin subunits.

We established that low basal levels of FAK Y397 phosphorylation in normotensive SD rat mesenteric arteries are predominantly associated with the β3 integrin subunit and that active c-Src associates with integrin α5β1. FAK Y397 phosphorylation was significantly increased in hypertensive TGR(mRen2)27 resistance arteries and recruited to FA sites after blood pressure rose and once eutrophic inward remodeling was complete. In addition, both pFAK Y397 and active c-Src associated with β1 and β3 integrins in the resistance arteries of TGR(mRen2)27 animals. However, the inhibition of remodeling by the administration of cRGDfV peptides resulted in the hypertrophy of VSMCs and a further increase of FAK Y397 phosphorylation. This process was associated with integrins containing the β1 subunit only.

We can conclude from these data that FAK phosphorylated at tyrosine position 397 (the autophosphorylation site) is present at both the integrin β1 and β3 cytoplasmic sites in the resistance arteries of hypertensive TGR(mRen2)27 animals, in contrast to SD controls, which exhibit relatively low pFAK Y397 levels only associated with the integrin β3 subunit.

We cannot rule out the possible involvement of FAK Y397 phosphorylation in subsequent length/autoregulation processes during the hypertension remodeling processes downstream of the integrin β3 subunit. However, it is now thought that the initial role, in the light of the FAK Y397 phosphorylation observed, is in mechanotransduction, as cRGDfV peptides not only inhibited eutrophic remodeling but also encouraged further FAK Y397 phosphorylation during the onset of hypertension in this model. Therefore, the increase of FAK Y397 phosphorylation observed is more likely to occur as a consequence of increases in wall tension, rather than acting as a direct intermediate of eutrophic inward remodeling. Besides aiding adhesion and migration [26], β1 and β3 integrins are both responsible for the intracellular transfer of these forces in the VSMCs of resistance arteries [27,28]. This conclusion is strengthened further by how the arterial segments of the cremaster muscle, at a high pressure (120 mm Hg), exhibit a prompt induction of FAK Y397 phosphorylation (<1 h) when compared with vessels pressurized at much lower levels and completely independent of neurohumoral influences [29].

There is the possibility that the increase of FAK Y397 phosphorylation observed in the resistance arteries of the young TGR(mRen2)27 hypertensive animals was induced by enhanced activity of the renin-angiotensin system (RAS; reviewed in [30]). However, this seems unlikely because at 5 weeks of age the resistance arteries of these animals have eutrophically remodeled without evidence of hypertrophy [6], similar to the morphological adaptations observed in a low-renin model of hypertension [31] and the BMH-2 mouse model which develops hypertension independently of the RAS [32]. The hallmark of a locally activated vascular RAS in older animals is vascular hypertrophy [30,33]. Again, ex vivo pressurization has determined that FAK Y397 phosphorylation is likely to be associated with an increase in pressure, in an Src-dependent manner, without the possible interference of neurohumoral influences. However, we cannot completely rule out the contribution of RAS activity to FAK Y397 phosphorylation in the TGR(mRen2)27 resistance arteries [34,35].

Various studies have reported the association of FAK with different β integrin subunits; β1, β3 and β5 cytoplasmic domains all contain sufficient information to trigger FAK phosphorylation and these specific regions have been identified [36]. Different cellular systems in vitro utilize different integrins for FAK activation, e.g. β3-but not β1-integrin engagement in cultured cardiomyocytes and VSMCs is accompanied by FAK activation in FAs [14,37]. In contrast, fibroblasts cultured on collagen employ β1 integrins only for FAK activation [38]. In response to the hepatocyte growth factor, VSMCs are known to utilize both β1 and β3 integrins for FAK activation and subsequent migration [39]. In this study, using the TGR(mRen2)27 rat model [16], it became apparent that both β1 and β3 integrins are involved in VSMC FAK recruitment/signaling when pressure becomes elevated, in contrast to normotensive controls. However, the FAK Y397 phosphorylation observed which is associated with β-integrin subunits in response to elevated pressure is not exclusively increased when eutrophic remodeling processes take place, but is already apparent at the initial stages when pressure becomes elevated.

## Clinical Perspectives

**Fig. 9:**
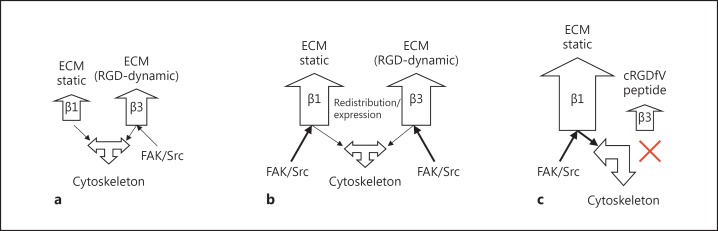
Schematic representation of the role of β integrins in mediating FAK Y397 autophosphorylation of the resistance arteries. **a** Normotenisve: integrin α5β1 (involved in calcium influx) and β3 integrin subunits are mechanically activated resulting in FAK Y397/Src Y418 phosphorylation, and are probably required for normal vascular integrity during normotensive pressure fluctuations. Relatively little change in FAK Y397 and Scr Y418 phosphorylation is required for normal vascular adaptations to minor changes in pressure. Signaling of FAK/Src via ‘general’ ECM-anchoring β1 integrins is minimal. **b** Hypertensive inward remodeling with cRADfV: during hypertensive remodeling, an increase in phosphorylation of the mechanosensitive FAK Y397/Src Y418 signaling complex is mediated via β1 and β3 integrins (but not the pSrc Y418 component of α5β1), suggesting a role of β1 and β3 integrin subunits to maintain vascular integrity in response to an increase in pressure. **c** Hypertrophic remodeling: abrogation of αVβ3 signaling with cRGDfV results in hypertrophy in response to pressure and mechanosignaling exclusively through β1 integrins. The majority of the β1 integrins are thought to be important in ‘anchoring’ VSMCs within the matrix of the vascular wall. Exclusive mechanosensing of FAK Y397 via β1 integrins through this rigid scaffold is thought to be central to these structural adaptations in hypertension.

It has been established that both β1 and β3 integrin subtypes are required not only in the adhesion of VSMCs to the ECM of the vascular wall but also for pressure-mediated signaling, resulting in vascular myogenic constriction and remodeling in hypertension [6,27] (summarized in fig. 9). In this study, we identified FAK Y397 phosphorylation as an early event as a result of elevated pressure in vivo and ex vivo. The identification of FAK Y397 phosphorylation in an Src-dependent manner is an important first step in resolving the complex signaling cascades that underlie integrin-mediated vascular adaptations of resistance arteries in hypertension. The relevance of unraveling molecular components in specific signaling events, in the context of pressure mechanotransduction in hypertension, will enlighten the design of pharmacological agents to prevent hypertension-mediated target-organ damage.

Recent data have suggested that the morphological changes in the arterial wall of small blood vessels are associated with cardiovascular prognosis. The normal response to untreated hypertension is eutrophic inward remodeling, i.e. a rearrangement of the vascular wall architecture without any evidence of growth [17]. When hypertrophy supervenes, this heralds the breakdown of normal homeostatic mechanisms such as autoregulation and, as such, there is clear evidence that this is the structural alteration associated with an adverse prognosis [3].
